# The impact of a training programme incorporating the conceptual framework of the International Classification of Functioning (ICF) on knowledge and attitudes regarding interprofessional practice in Rwandan health professionals: a cluster randomized control trial

**DOI:** 10.1186/s12909-021-02537-7

**Published:** 2021-03-01

**Authors:** Jean Baptiste Sagahutu, Jeanne Kagwiza, Francois Cilliers, Jennifer Jelsma

**Affiliations:** 1 College of Medicine and Health Sciences, University of Rwanda, Kigali, Rwanda; 2 University of Cape Town, Cape Town, South Africa

**Keywords:** Interprofessional, ICF, Rwanda, District hospital

## Abstract

**Background:**

The first step in improving interprofessional teamwork entails training health professionals (HP) to acknowledge the role and value the contribution of each member of the team. The International Classification of Functioning, Disability and Health (ICF) has been developed by WHO to provide a common language to facilitate communication between HPs.

**Objective:**

To determine whether ICF training programme would result in improved knowledge and attitudes regarding interprofessional practice within Rwandan district hospitals.

**Design, setting and participants:**

A cluster randomised, single blinded, control trial design was used to select four district hospitals. Participants included physicians, social workers, physiotherapists, nutritionists, clinical psychologists/mental health nurses.

**Intervention:**

Health professionals either received one day’s training in interprofessional practice (IPP) based on the ICF (experimental group) as a collaborative framework or a short talk on the topic (control group).

**Outcome measures:**

Validated questionnaires were used to explore changes in knowledge and attitudes. Ethical approval was obtained from the relevant authorities.

**Results:**

There were 103 participants in the experimental and 100 in the control group. There was no significant difference between Knowledge and Attitude scales at baseline. Post-intervention the experimental group (mean = 41.3, SD = 9.5) scored significantly higher on the knowledge scale than the control group (mean = 17.7, SD = 4.7 (t = 22.5; *p* < .001)). The median scores on the Attitude Scale improved in the Experimental group from 77.8 to 91.1%, whereas the median scores of the control remained approximately 80% (Adjusted Z = 10.72p < .001).

**Conclusion:**

The ICF proved to be a useful framework for structuring the training of all HPs in IPP and the training resulted in a significant improvement in knowledge and attitudes regarding IPP. As suggested by the HPs, more training and refresher courses were needed for sustainability and the training should be extended to other hospitals in Rwanda. It is thus recommended that the framework can be used in interprofessional education and practice in Rwanda and possibly in other similar countries.

**Trial registration:**

***Name of the registry:*** Pan African Clinical Trial Registry.

***Trial registration number:*** PACTR201604001185358.

***Date of registration:*** 22/04/2016.

***URL of trial registry record:*** www.pactr.org

## Background

The Framework for Action on Interprofessional Education and Collaborative Practice which was established for all countries’ policy makers, health educators and health workers reported that several countries throughout the world are struggling to maintain their health systems and cannot address the problems that are emerging with health care delivery [54, 55]. Problems identified by Frenk et al. [19] included poor teamwork and the “so-called tribalism of the professions” (amongst others).

It is estimated that a patient, during his/her period of about four days hospital stay can be seen by around 50 different people including medical doctors, nurses, physiotherapists, and others [12]. Apart from duplication of effort, poor collaboration between health care professionals can result in medical errors, lack of critical information and inadequate interpretation of health information, all risks to a patient’s safety [12]. The Lancet Global Independent Commission therefore identified the need for health care reform based on interprofessional and transprofessional education that breaks down professional silos while enhancing collaborative and non-hierarchical relationships in effective teams [19].

Interprofessional collaboration occurs when health professionals from different disciplines with different backgrounds provide patient care together and work closely with each other and the patient, family and the community to deliver the optimum health care [21, 28, 53]. Smooth collaboration among health care providers may minimise medical errors and eventually improve patient outcomes [18]. A framework of outcome measures for effective teamwork [30], a systematic review on the effects of interdisciplinary team care interventions on general medical wards [33], and A Cluster Randomised Control Trial (CRCT) conducted by Borenstein et al. [7] among an elderly population demonstrated the positive effect of interprofessional communication for better collaborative practice. These include reduced hospitalization time and cost, reduced anticipated admission, better accessibility and improved coordination of care, efficient use of healthcare services, reduced medical errors and improved health outcomes, and great role clarity. Despite the potential benefits and effect of interprofessional communication and collaborative practice, there are also some challenges when professionals from various disciplines work together. These include different goals and priorities among disciplines, misunderstanding of others’ responsibilities and roles, and overlapping of some professions’ roles during care delivery, lack of recognition of each professional’s expertise, resistance to innovation, professional hierarchy, lack of integrative skills, lack of enough time, lack of space, and lack of training programmes [9, 35, 43, 48]. However, a interprofessional framework which should guide communication and collaboration between health care professionals in their clinical settings is therefore needed.

In 2001, the WHO produced the International Classification of Functioning, Disability and Health (ICF), a member of the International Family of Classifications and a sister classification to the universally used International Classification of Disease (ICD). The ICF presents a common and standardised language for the description of health and health-related states [52]. The six interactive components of ICF are structured in two main parts [47, 51]. Part one covers information related to body functioning and disability, while part two deals with contextual factors [51, 54]. Each part also has two components: functioning and disability (body functions and body structures; activities and participation); contextual factors (environmental factors and personal Factors) [51, 54]. The ICF does not present the process of functional limitations as linear and instead posits an interaction between the health condition, functioning and environmental factors, which in many cases may be reciprocal. *Body structures* reflect anatomical parts of the body (e.g. legs, arms, etc.) and also organs (e.g. heart, brain). On the other hand, *body functions* reflect physiological functions of the human body (e.g. psychological function, mental function, voluntary movements). Therefore, any problem in body structure or function relates to the impairment [51, 54]. *Activities* refer to the execution of actions or tasks by an individual. If someone has difficulties in executing the usual tasks or actions such as walking, sitting, eating and washing, he/she will have *activity limitation* [51, 54]. *Participation* is involvement in life situations by individuals such as leisure, work, family and community, and school activities. Therefore, if a person encounters problems hindering him/her to be engaged in those activities as usual, he/she has a *participation restriction* [51, 54]. *Environmental factors* are physical, psychological and social (psycho-social) environments where a person performs his or her usual activities or lives [1, 51]. The environmental factors can include a variety of areas such as home, work, policies and legislation, family and community, friends, medications and assistive devices. Environmental factors have an impact upon an individual’s functioning as facilitators or barriers [54]. Personal factors imply how a person experiences a particular health condition. These include age, gender, education, profession, social background, individual lifestyle, personal character and coping strategies [51].

Indeed, the ICF, by its nature, is multifaceted to be used by different health.

Professionals [22]. The ICF also represents a paradigm for approaching health and health care, and demonstrates the need for different health care professionals. Therefore, it encourages health care professionals to consider health issues underlying their scope of practice [13]. In interprofessional practice, the ICF framework contributed a lot to the assessment of complex health conditions that should not be treated by one profession [2]. Based on its nature that tackles different aspects of health, the ICF provides a useful functioning assessment among health care professionals, who basically used the biomedical model of assessment, for identifying all relevant information related to the condition [47]. In this way, the ICF improves communication among health care professionals during patient assessment by providing the universal language and framework for health and functioning [2, 16]. Furthermore, the use of ICF as a tool for interprofessional collaboration improves the quality of the work process through the systematic assessment approach, settings treatment goals and plans [38]. This implies that team work is promoted by using a shared language in the patient management process in either education or practice in the hospital environment [13, 24]. In fact, the ICF was found to be an effective instrument for decision making, collaboration and communication among health professionals from various disciplines due to its common language across the globe [31].

However, the application of the ICF is as yet somewhat limited among health professionals, especially those who are not part of a rehabilitation team [8, 49]. In addition, there are limited published accounts of including the ICF in the training of health professionals in IPP. (The ICF is premised on the biopsychosocial approach to health care. This is in contrast to health care in many countries, including Rwanda, where the bio-medical approach is the dominant model and lack of collaboration between health care professionals in different disciplines.

The recent study on the impact of a training programme incorporating the ICF framework has suggested the improved behaviour regarding interprofessional practice in Rwandan health professionals demonstrated by quality of medical recording [42]. Various studies demonstrated that change in behaviour may be influenced by the change in knowledge and attitudes [11, 17, 29]. As suggested by the Global Independent Commission, strengthened IPP might well result in improved patient outcome. However, there is little evidence within the African context as to the most effective strategies to strengthen IPP within a hospital setting. The aim of this study was therefore to explore whether the presentation of a day’s workshop on IPP, based on the biopsychosocial model of health care as formulated in the ICF framework, would result in improved attitudes and knowledge of IPP.

## Methods

The study was carried out in district hospitals in Rwanda. Rwanda has 40 district hospitals covering four provinces plus four district hospitals in Kigali city to make a total of 44 district hospitals [41]. In general, apart from Kigali city, all district hospitals in Rwanda are similar in terms of patients and conditions, services, materials and equipment as well as health care personnel, so the four hospitals from Kigali were excluded from this study. The district hospitals have both inpatient and outpatient services.

The current study is part of a study published recently by Sagahutu et al. [42] which investigated the impact of a training programme incorporating the conceptual framework of the International Classification of Functioning (ICF) on behaviour regarding interprofessional practice in Rwandan health professionals. Therefore, this study only focused on the impact of the ICF training on the knowledge and attitudes regarding interprofessional practice using pre and post data. A Cluster Randomised Control Trial (CRCT) with single blinding using a pragmatic study design was used in this study to select the district hospitals and allocate either to experimental or control arm. This design is primarily used to avoid contamination between the control and experimental groups when a single setting (such as a ward) is utilised [23]. Four of the 40 district hospitals were randomly allocated to receive a day’s training in interprofessional practice using the ICF (experimental hospitals) or only a short talk (introduction) between one and two hours on the ICF (control hospital) by a blinded research assistant. Pre- and post-measurements of knowledge and attitudes towards IPP were performed at baseline and after training. A two hour face to face meeting was held with the experimental group two months later to receive feedback on the implementation of the workshop recommendations. The entire study adheres to CONSORT guidelines.

The sample targeted all health care workers employed within four randomly selected hospitals including Medical doctors, Physiotherapists, Nurses, Social Workers, Mental Health Nurses/Clinical Psychologists, and Nutritionists. These health care professionals most commonly provide ward service in the district hospitals of Rwanda. The inclusion criteria were licensed full time health professionals, working either in orthopaedic/surgical, medical or paediatric wards, with at least six months of working experience. The primary outcome of this study was the improved knowledge and attitudes regarding the interprofessional collaborative practice among health professionals.

A feasibility study was undertaken and based on these results we anticipated a large effect size on the knowledge and attitudes questionnaires. Using the parameters identified by the pilot study and allowing for cluster sampling and a design effect of 1.5, the effective sample size (ESS) required a 10% sample of the 40 district hospitals (four hospitals) with 64 participants. The ESS is the sample required to reach adequate power, once the design effect has been taken into account. The design effect is a measure of the correlation between subjects in each cluster. The more similar they are with regard to the outcome of interest, the greater the design effect and the smaller the ESS becomes. The design effect, or inflation factor is 1 + (*n −* 1) *p,* Where *n* = the number per cluster and *p* or rho is the intra-cluster correlation (ICC). Killip and Mahfoud [26] reports that in human studies values of ICC rho fall between 0.01 and 0.02 and we thus used a rho of .02. Using STATISTICA, we calculated that, in a simple RCT, a sample of 16 would be necessary in each group if we anticipated that the control group would improve their scores in the knowledge questionnaire to 15 and the experimental up to 29 with the SD gained from the pilot study of 11.8 (based on the feasibility study results). The *p* value (Alpha) was set at .05, and the power (Sigma) at .9.

**Table:** 

	Value
Population mean post intervention of control group	15.00
Population mean post intervention of experimental group	29.00
Population S.D. (Sigma)	11.80
Standardized effect (Es)	−1.19
Type I error rate (Alpha)	0.05
Critical value of t	2.04
Power goal	0.90
Actual power for required N	0.90
Required N (per group)	16.0

If we then enter these values into the equation to calculate the ESS, which is N*number of clusters/design effect [26], we get (16*4)/ (1+ (16–1)*.02) =48. In other words, to be adequately powered, the study would need a total of 64 participants but due to the design effect, the ESS becomes 48. The sample at each hospital was increased to 50 which would thus give an ESS of 100 to ensure adequate power. Two hospitals were randomly allocated to the experimental arm and two other hospitals to the control arm by a blinded research assistant.

### Instrumentation

Despite the extensive literature and questionnaire search, no standardised questionnaire was found to measure the intended construct in knowledge of health care professionals on interprofessional practice using the ICF framework. A self-designed questionnaire was developed to monitor the *knowledge* of health workers of interprofessional practice and the ICF. An integrated case study was designed taking into account the role of different professionals in assessment and management of patient. The questionnaire was developed considering each ICF domain under which the participant should indicate the profession to intervene and the probable required intervention. The questionnaire was composed of three parts: *Part one* consisted of demographic information such as gender, age, profession, ward/department, level of education and years of experience. Part two presented an integrated case study to inform the answers of the 3rd part. Part three included questions relating to the case study. The respondents were requested to identify problems under each of the ICF domains and then to list the professions that should be involved in management of the condition and the possible intervention that they could give. Each of the correct responses was awarded one mark. Apart from the patient conditions, other items of the questionnaire relate to impairment, current activity limitations, activity limitations anticipated on discharge, participation restrictions, positive and negative personal factors, and environmental factors (facilitators and barriers).

The Attitudes questionnaire was based on the Attitudes Towards Health Care Teams (ATHCT) scale modified by Leipzig et al. [27] into a 21-item tool with three subscales: Attitudes Toward Team Values (11 items), Attitudes Towards Team Efficiency (5 items), and Attitudes Towards Physician’s Shared Role on Team (5items). The 21-item scale was tested in the feasibility study in Rwandan Hospital setting.

The content validity index (CVI) was calculated based on the responses of three experts who were purposively recruited based on their expertise and availability. The content validity index (CVI) was calculated and the internal consistency and effect size were determined. The final versions of the two questionnaires demonstrated excellent overall scale content validity index (S-CVI) (Knowledge questionnaire: S-CVI = .97; Attitudes Scale: S-CVI = .95). The inter-rater reliability was tested, for the knowledge questionnaire, where the intra-class correlation for absolute agreement was .976 (Confidence Intervals (CIs) = .962, .987). The internal consistency for the Attitudes Scale was (Cronbach’s Alpha = .76).

A model of the transfer of training process by Grossman and Salas [20] adopted from Baldwin and Ford (1988) [4] was used. The sessions were held within the hospitals’ facilities (work environment) to ensure maximum relevance to daily work. The content of the training programme was based on literature related to best practice in continuing professional development, faculty development and transfer of training. It was also developed based on the experience from Stellenbosch University through the Centre for Health Professions Education. Drawing on the experience of other programmes, different methods were employed such as participatory lectures, practical sessions, and role playing. After training, the post-test measurements on knowledge and attitudes were obtained. The training content and full trial protocol are available from the corresponding author on request. The control hospitals received no formal training during this time, but they were given a basic introduction to the ICF framework in a didactic lecture which took between one to two hours.

### Procedure

Permission to conduct the study was obtained from the relevant institutions and participants. Ethical approval was sought from the University of Cape Town, Faculty of Health Sciences Human Research Ethics Committee (Ref: HREC/REF: 085/205) and the National Health Research Committee (Ref: NHRC/2015/PROT/016) of Rwanda. The Pan-African Clinical Trial Registry number is PACTR201604001185358. Signed informed written consent was obtained from each health professional who participated.

The intervention was novel and we were unsure whether there would be adequate recruitment and compliance with the study. Therefore a feasibility study with 60 health professionals was carried out at a fifth district hospital which is similar to the settings of the hospitals in the main study but not included in the main study. The pilot indicated that the project would be feasible but that the planned duration of the training (two days) was too long. As a result, the programme was shortened to one day. Other suggestions such as focusing more on case studies and real patients’ records were also implemented. The feasibility study also assisted in the determination of sample size.

After obtaining ethical clearance and permission from the hospital superintendents, the researcher had meetings with the medical chief of staff and chief of nurses to plan the intervention. This included selection of participants to be invited, days of training, allocation of participants to different days in order to maintain the clinical work within the hospitals, and the venue and all logistics needed during training. Participants were divided in three groups (group one to be trained on day one, group two on day two, and group three) to accommodate and recruit at least 50 health professionals at each hospital.

### Brief content and description of training

The getting to know one another play combined with describing the own professional role and that of another was the first item of the training*.* In this activity, the participants explored the roles and responsibilities of different professions. Using the case of a diabetic patient, every participant had to pick the profession which was different from his/her profession. In small groups, the participants wrote everything he/she perceived the profession he/she represented could do for that particular case, bearing in mind the possible complications of the case. They were requested not to talk about their own profession or make corrections.

*Clearing misconception:* in a large group, the participants presented what they perceived to be the role of other professionals in case management. After presenting each professional’s role, the participants from that profession clearly described the role of their profession in managing the given case. Before lunch, the ICF framework was presented as the framework to provide a common language between health care professionals. After lunch, three patient records were randomly selected and three small groups were formed to be given on patient record making sure that there were mixed groups of different professions. They were requested to identify the problems that were addressed in the patient records and probable problems that were not addressed. Each group had time to present. In plenary, based on the discussed patients’ records, the trainer asked the participants: what are the facilitators and barriers to implementing the interprofessional practice in your hospital? How can you use those facilitators for implementation? How can you address those barriers to implementation?

It was arranged in such a way that each discipline was represented in each group. The participant information letters were provided and consent was obtained from participants who agreed to participate. The same activities were done in the control hospitals.

On the day of training, the pre-test knowledge and attitudes measurements were performed prior to the start of the programme. The two hours ICF introduction in the control group was conducted in one session at each hospital during their morning staff meeting.

In order to facilitate analysis, some of the items in the attitudes questionnaire, which were framed in such a way that a negative response (strongly disagree) was the desirable response, were inverted so that strongly desirable responses were all 5 and strongly undesirable responses were all 1. The analysis was performed by IBM SPSS (version21) and STATISTICA (Version 13.2 DELL INC). Descriptive statistics were used to describe the characteristics of the health care professionals and the scores on the different outcome measures. The experimental and control groups were compared at baseline to ensure equivalence between the demographic characteristics of the participants. The Chi-square test was performed to determine whether there was an association between gender, profession, place of work and group (experimental and control). For continuous variables parametric tests, such as the independent t-test and ANOVA, were used to compare the two groups. As the attitudes scale yielded ordinal data, the Mann-Whitney U test was used to establish if the two sets of groups were equivalent before and after training and Kruskal Wallis test was used for rankings of the scores of different professions before and after the intervention. A significance level (α) of 0.05 was used throughout the study. The data were presented graphically in the form of tables, box plots, and graphs.

## Results

A total of 203 health professionals participated in pre and post measurement, ranging from 50 to 53 participants in each hospital. The mean age was 35.7 years (SD = 8.29) and there was no difference in age between the two groups (*p* = .208). There was no association between gender, profession, place of work and group. However, the experimental group had a significantly greater number of years of experience (*p* = .030). The proportion of medical doctors who were eligible to attend and did so was lowest of all the health professionals, apart from the mental health workers. A slightly greater percentage of the control group personnel attended (91% compared to 88%). (Table 1).

**Table 1:** Participants through the study across the groups and professions at baseline

Group	Profession	Eligible	Baseline	% attending
Experimental	Medical doctors	10	5	50.0
	Nurses	87	83	95.4
	Physiotherapists	6	5	83.3
	Social workers	6	5	83.3
	Mental health nurses	4	2	50.0
	Nutritionists	4	3	75.0
	Total Experimental	117	103	88.0
Control	Medical doctors	9	6	66.7
	Nurses	84	80	95.2
	Physiotherapists	4	4	100
	Social workers	5	5	100
	Mental health	3	3	100
	Nutritionists	2	2	100
	Total Control	107	100	93.5
Total		224	203	90.6

Percentages are included to allow for comparison, despite the small numbers

### Knowledge

As can be seen below, there was no significant difference in the mean percentage of correct responses for any section in the Knowledge Questionnaire or in the overall mean percentage of correct responses to the scale on the Pre-intervention tests. The sections which scored highest included items related to impairments of body structure and function (50% correct). This was followed by the activity (17%) and negative personal factors (13%) sections.

Post-intervention testing indicated that there was a highly significant difference between the groups in knowledge in all items and in overall score (*p* < .001). The difference in the mean total scores between the groups was was 23.6% at post measurement with the experimental group showing the greatest improvement. There was also a significant improvement within group performance in both the experimental and the control group. The experimental group improved 29% and the control group improved 5%. (Table 2).

**Table 2:** Comparison of knowledge by group

	Experi-mental		Control			
Pre-intervention: Scale Section	Mean %^a^	S.D	Mean %^a^	S.D	t-value	p
Health condition	49.6	16.8	47.4	14.6	1	0.32
Impairment	8.2	15.3	11.7	12.4	−1.8	0.077
Activity limitation (Current problems)	17.4	14.2	15.7	11.5	0.9	0.351
Activity limitation (Problems anticipated on discharge)	9.5	16	11.4	13.7	−0.9	0.350
Participation restriction	4	6.8	4.5	5.6	−0.5	0.621
Personal factors (positive)	5.5	10.7	6.7	10	−0.8	0.422
Personal factors (negative)	13.4	16.3	13.7	14.5	−0.1	0.910
Environmental factors (facilitators)	7.2	11.1	6.8	9.1	0.3	0.783
Environmental factors (barriers)	5.5	8.4	6.4	8.1	−0.8	0.450
Mean % correct for whole scale	12.4	6.4	12.8	5.0	−0.5	0.611
Post-intervention: Scale Section						
Health condition %	68.9	11.4	56.8	9.6	8.2	<.001
Impairment %	44	20	20.8	16.1	9.1	<.001
Activity limitation (Current problems) %	38.6	14.8	21.2	9.9	9.9	<.001
Activity limitation (Problems anticipated on discharge) %	52.6	28.1	18.8	13.7	11	<.001
Participation restriction	29.8	14.4	7.3	5.4	14.9	<.001
Personal factors (positive)	37.1	21.8	13	11.2	10	<.001
Personal factors (negative)	42	22	14	14.4	10.7	<.001
Environmental factors (facilitators)	45.5	20.4	10.6	10.9	11.3	<.001
Environmental factors (barriers)	33.8	17.3	7.4	7.5	14.2	<.001
Mean % correct for whole scale	41.3	9.5	17.7	4.7	22.5	<.001
Within group comparison	Pre		Post		t value	p
	Mean %^a^	S.D	Mean %^a^	S.D		
Experimental group	12.4	6.4	41.3	9.5	33.82	<.001
Control group	12.8	5.0	17.7	4.7	13.13	<.001

^a^% of responses correct

**Fig. 1:**
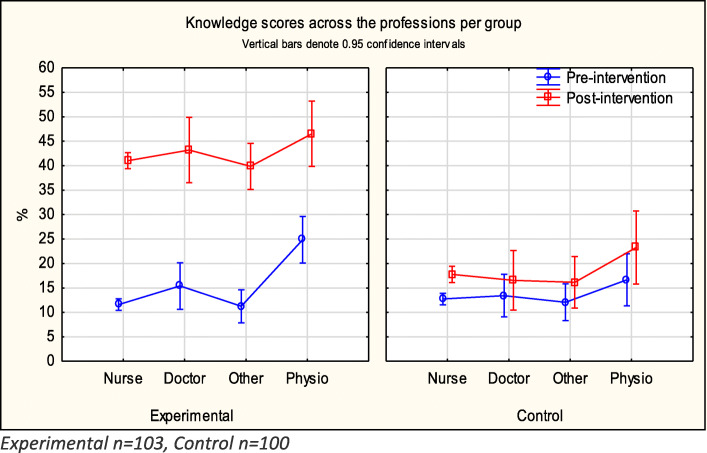
Knowledge scores across professions per group

Due to the relatively small numbers in each group, apart from Nursing, the 95% confidence intervals were large and statistical analysis was not undertaken. Physiotherapists scored the highest in all groups, both pre- and post-intervention, followed by medical doctors in the experimental group. The increase in knowledge in the experimental group was evident across all professions post-intervention. Although the control group also improved, the 95% confidence intervals pre- and post-intervention scores overlapped for all professions apart from the Nursing. (Fig. 1).

Pre-intervention knowledge scores show that physiotherapists scored higher than other professionals in both the experimental and control groups.

The correlation between the pre- and post-intervention scores was rho = 0.353 in the experimental group and rho = 0.396 in the control group, both significant at a *p* < .01 level.

### Attitudes

The level of agreement was almost the same between two groups before intervention, but after intervention the level of agreement was higher in experimental compared to the control group. The median scores of the experimental group improved from 77.8 to 91.1%, whereas the median scores of the control group remained approximately 80%. (Table 3).

**Table 3:** The pre- and post-training responses to the attitudes questionnaire

Items	Exp*	**1	2	3	4	5	Con*	1	2	3	4	5
1.Working in teams does not unnecessarily complicate things most of the time	Pre	4	15	13	27	44		5	15	17	30	34
	Post	0	1	9	24	69		0	3	17	32	49
2.The team approach improves the quality of care to patients	Pre	0	9	15	35	44		0	10	9	39	43
	Post	2	1	0	12	88		1	13	5	31	51
3.Team meetings foster communication among team members from different disciplines	Pre	2	4	16	36	45		3	3	11	40	44
	Post	2	0	0	17	84		1	5	3	36	56
4.Patients receiving team care are more likely than other patients to be treated as whole persons	Pre	3	7	20	38	35		2	3	19	43	34
	Post	1	0	0	20	82		4	2	5	40	50
5.A team’s primary purpose is not to assist physicians in achieving treatment goals for patients	Pre	10	14	30	23	26		3	18	25	24	31
	Post	0	2	10	35	56		3	9	12	28	49
6.Working in a team keeps most health professionals enthusiastic and interested in their jobs	Pre	1	8	14	41	39		0	6	13	47	35
	Post	1	0	2	13	87		6	4	16	36	39
7.Patients are highly satisfied with their care when it is provided by a team	Pre	4	12	17	26	44		1	16	14	35	35
	Post	0	1	11	20	71		11	14	7	30	39
8.Developing a patient care plan with other team members avoids errors in delivering care	Pre	2	5	12	27	57		1	1	13	35	51
	Post	3	0	0	10	90		6	8	15	26	46
9.When developing interprofessional patient care plans, not much time is wasted translating jargon from other disciplines	Pre	2	12	30	29	30		0	15	13	34	39
	Post	1	0	4	32	66		2	5	4	39	51
10.Health professionals working in teams are more responsive than others to the emotional and financial needs of patients	Pre	9	10	19	38	27		6	7	17	44	27
	Post	0	3	9	34	57		3	5	11	41	41
11.Developing an interprofessional patient care plan is not excessively time consuming	Pre	4	9	29	30	31		0	8	23	25	45
	Post	0	4	12	40	47		0	5	9	30	57
12.The give and take among team members helps them make better patient care decisions	Pre	4	3	8	36	52		2	2	10	44	43
	Post	1	1	2	27	72		15	13	5	32	36
13.In most instances, the time required for team meetings could not be better spent in other ways	Pre	4	16	22	34	27		3	12	19	38	29
	Post	1	2	13	32	55		1	7	14	36	43
14.The physician does not have the ultimate legal responsibility for decisions made by the team	Pre	6	15	24	34	24		2	9	26	29	35
	Post	0	0	14	30	59		3	4	18	27	49
15.Hospital patients who receive team care are better prepared for discharge than other patients	Pre	8	8	10	33	44		7	6	13	38	37
	Post	0	1	6	24	72		14	13	11	27	36
16.The team approach makes the delivery of care more efficient	Pre	1	4	3	40	55		4	4	5	42	46
	Post	3	3	9	27	61		13	8	9	28	43
17.The team approach permits health professionals to meet the needs of family caregivers as well as patients	Pre	4	8	12	36	43		5	5	13	42	36
	Post	0	2	11	26	64		5	13	16	31	36
18.Having to report observations to the team helps team members better understand the work of other health professionals	Pre	1	8	9	44	41		1	1	13	43	43
	Post	1	0	2	40	60		11	5	13	33	39

Exp* = Experimental Group; Con* = Control Group; 1 = strongly disagree; 2 = Disagree, 3 = Neutral, 4 = Agree, 5 = Strongly Agree

There was no significant difference in the ranking of the scores on the attitudes scale of two groups prior to training). However, after training the experimental group scored significantly higher (Adjusted Z = 10.72, *p* < .001). (Table 4).

**Table 4:** Comparison of between and within group differences in the sum scores of the attitude scale across the group

Between group (Mann Whitney U)	Rank Sum Experimental	Rank Sum Control	U	Z Adjusted	p value
% Pre-intervention	9861	11,050	4505	−1.66	0.098
% Post-intervention	15,070	5840	689	10.72	<.001
Within Group (Sign test)	N of non-ties	% Post-test higher than pre-test		Z Adjusted	p value
Experimental group	103	98.06		9.66	<.001
Control group	93	59.14		1.66	.097

Experimental N = 103, Control N = 100

**Fig. 2:**
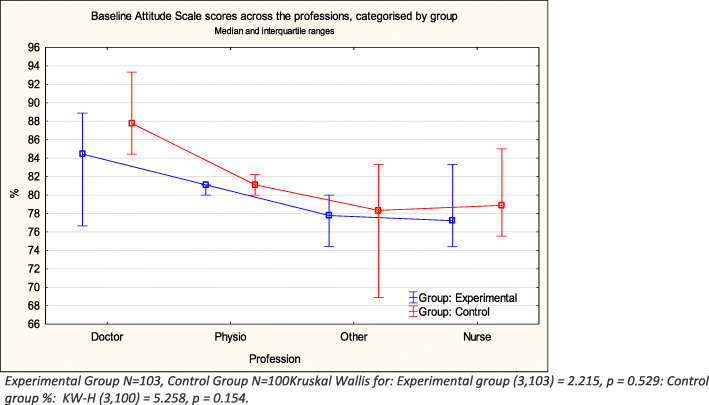
Pre-intervention attitude scale scores professions, categorised by group

**Fig. 3:**
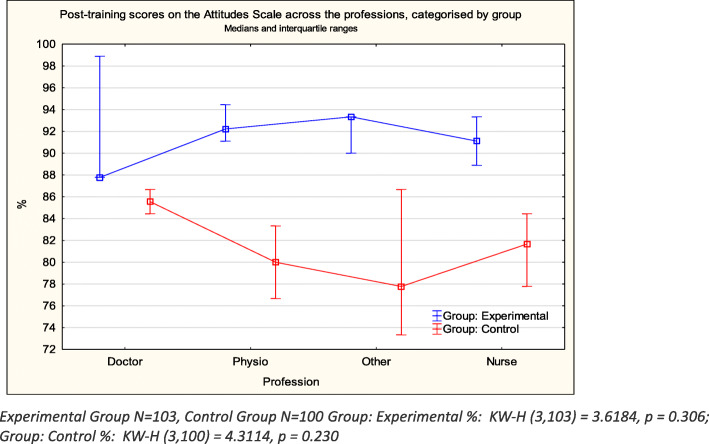
Post-intervention attitude scale scores by professions

The interquartile ranges were large and there was no significant difference detected by the Kruskal Wallis ANOVA between the scores of the different professions in either group, either before or after the intervention. However, Figs. 2 and 3 clearly indicate that, whereas the pattern of scoring remained the same in the control group, with medical doctors scoring the highest both pre- and post- test, the pattern changed considerably in the experimental group post-training. Whereas the doctors scores remained similar, the other three professions showed considerable improvement and all scored higher than the doctors on post-testing. (Figs. 2 and 3).

## Discussion

This study provided evidence that a one day training programme can result in a large improvement in both the knowledge regarding the ICF and IP and attitudes towards teamwork within the Rwandan context. To the best of our knowledge, this is the first study on the use of ICF as a theoretical framework to inform interprofessional assessment and management by health care professionals in a hospital setting within a low to middle income country. There are studies, in other countries, that utilised the ICF to inform interprofessional practice in different health conditions, but these were mostly within academic rather than clinical settings [8, 45, 46]. Furthermore, the majority of studies using ICF in interprofessional care were conducted in rehabilitation settings and in high income countries [37, 47, 49]. However, studies involving interprofessional training using ICF as a communicating framework in hospital settings in resource constrained environments are scarce.

### Participants

The willingness of district hospital staff to participate was encouraging and 91% of eligible health professionals were recruited, with all disciplines included. Several of those not attending were reported anecdotally to be on leave or have other concerns. The approval granted by the local Human Research Ethics Committee and the support of both the central and local health authorities played an essential role in gaining buy-in from the health professionals. Rwanda has a relatively small pool of health professionals and the high recruitment may be a reflection of the influence that authorities have over the decision making of those employed in the public sector and possible peer pressure. There is a strong commitment to improved health care and the rebuilding of the health care system after the genocide [32, 39] and the high recruitment may have been an indication that the Rwandan public health system is ready to embrace interprofessional teamwork as a means to that end. This is a likely explanation as several respondents, in training satisfaction questionnaire, suggested that the training be incorporated into the training of health professionals at all levels.

The respondents were also relatively young (35.7 years, SD = 8.29) as a large number of health care professionals were trained after the 1994 genocide. Unfortunately, a large number of health care professionals trained before the genocide, while others went in exile. Therefore, Rwanda has started to rebuild its health care system after the genocide [32, 39]. It was also observed that, in Rwanda, a number of health professionals upgraded their education level in other fields and changed their careers to something other than medical after some years of practice. Therefore, this explains the young age group of health care professionals working in Rwanda district hospitals. As younger people may be more responsive and open to new ideas [54]; the age of the participants could also have contributed to the success of the programme.

Although the proportion of medical doctors and mental health workers recruited was the lowest of all professions, at least 50% did participate. This lower rate of recruitment could have been a source of bias as the traditional hierarchical view may have led to resistance to attending sessions led by a physiotherapist. It is more likely that the lower recruitment rate reflects an increased work load (there were only 19 doctors in the four hospitals). In any case, the first step in breaking down the professional silos is to get the different health professionals talking to each other about issues related to interprofessional practice which was achieved in this study.

The sample thus appears to have been representative of health professionals in the selected hospitals. As a similar staffing complement is deployed to most district hospitals, it is likely that the results can be generalised to all district hospitals in Rwanda.

### Impact on knowledge

The pre-intervention results indicated a low level of knowledge regarding the ICF and of the holistic management of patients. The participants scored highest with regard to those aspects of patient management which are traditionally included in the examination of patients. These include the health condition (nearly 50% answered this section correctly), followed by functional problems with regard to limitations in activities and personal characteristics which would impact negatively on health. In contrast, those components which are part of a more holistic approach to management, such as activity limitations anticipated after discharge, participation and the impact of environmental factors were poorly understood. The pre-intervention results supported the original contention that health care in district hospitals in Rwanda adhere to the medical model of care, rather than a more holistic biopsychosocial approach.

It is noteworthy but not surprising that physiotherapists scored higher than other professionals. Most Rwandan physiotherapists were trained in the same institution (University of Rwanda) and are more likely to have some ICF knowledge from their education training as this is offered during the physiotherapy programme. Despite having had previous exposure, they still scored 25% or less, which would indicate that more time should be spent on the ICF and interprofessional practice during their basic training. These results do suggest, however, that the introduction to IPP and the ICF should be included in the undergraduate training curricula of health care personnel.

The intervention resulted in an improvement in both groups, but it was significantly larger in the experimental group. This implies that the provision of a lecture and handouts does result in an increase in knowledge, but that the full day training was more effective in bringing about change. These results were not unexpected. As the knowledge levels were low at base-line, there was much room for improvement. The literature also supports the effectiveness of training in improving knowledge of interprofessional practice [50]. Papers that have reported on effective knowledge transfer through training include those by Pless et al. [36] in their study evaluating the in-service training in using the ICF and ICF-CY and Phillips et al. [35] who demonstrated immediate improvement in knowledge after training across all questionnaire items; Ericson et al. [15] on interprofessional training in health care students; Bays et al. [5] on interprofessional communication in serious diseases; Bain et al. (2014) [3] on interprofessional training in chronic disease settings; and Zanotti et al. [57] in on-field training in interprofessional education among medical students. The positive impact of the two-hour ICF introduction intervention supplied to the control group should not be overlooked, as it may be a cost-effective method to introduce the district health teams to the concepts of interprofessional practice and the ICF.

Interprofessional collaboration could be enhanced by strategies like interprofessional ward rounds, education, clubs, and trainings on top [44]. The ICF framework provides a common language for biopsychosocial holistic patient care facilitates effective interprofessional collaboration [36]. By improving knowledge, interprofessional training can improve the collaborative health care team and the quality of care [56]. Furthermore, the improved knowledge after training may result in both a change in attitude and in practice [34].

### Impact on attitudes

The baseline scores of both groups were high before the intervention. Our findings were consistent with several studies which assessed the impact of interprofessional training in improving attitudes, although many were with health care students across disciplines [10, 14, 25, 40].

Although there were no significant differences detected between the scores of the different professions, this may have been as a result of the small sample sizes for some professions and the large inter-quartile ranges. However, it is interesting to note that, based on the plots of the pre- and post-intervention scores for each group, medical doctors scored highest in each group pre-intervention and showed the least improvement post-intervention. A study conducted by Jacobsen and Lindqvist [25] demonstrated the change in attitude in other professionals similar to our study, but the scores of medical doctors remained almost the same despite a two-week interprofessional training. The intervention appeared to be most effective for other professions and the lack of effect on medical doctors may due to the fact that they had high pre-intervention attitudes scores.

### Study limitations

Though the rigorous feasibility study which led to useful amendments to the protocol, the training and the outcome measures was performed prior to the main study, there are some limitations in this study. These include the level of participation among medical doctors and mental health nurses.

A further consideration is that, post-training socially desirable answers might have become apparent Beaulieu, Adrien, Potvin and Dassa [6] and this could have resulted in bias towards reporting improved attitudes without a genuine change taking place. This is a problem common to many attitudes scales and it was thus necessary to include the monitoring of behaviour change in addition to the knowledge and attitude changes.

## Conclusion

In conclusion, the intervention proved to be effective. The use of the ICF as a framework for training health professionals regarding IP resulted in a significant improvement in knowledge and attitudes regarding interprofessional collaborative practice.. This introduction of the ICF as the framework to inform interprofessional assessment and management in Rwanda could result in the adaptation of the biopsychosocial model and a more holistic approach to care. However, “deep” or fundamental improvement in knowledge and attitudes should be reflected in practice. It is proved to be relatively easy to change knowledge and reported attitudes through training intervention. Indeed, the real change should reflect improved behaviour or daily practice and this were examined in a recently published paper by Sagahutu et al. [42].

It is hoped that the findings of this study may contribute to improving health care delivery in Rwandan district hospitals and health system at large. It is thus recommended that the framework be used to promote interprofessional education in Rwanda and other similar countries. The bio-psycho-social approach to health care, including IPP, should be introduced in the training of health professionals. The ICF appears to be a useful conceptual framework in which to do this. However, there is a need for reinforcing the training over time for sustainability or retention of knowledge and attitudes.

## Data Availability

All data generated or analysed during this study are included in this published article [and its supplementary information files].

## Abbreviations

ATHCT: Attitudes Towards Health Care Teams scale
CVI: Content Validity Index
HREC: Human Research Ethics Committee National Health Research Committee
ICD: International Classification of diseases
ICF: International Classification of Functioning, Disability and Health
ICF-CY: International Classification of Functioning, Disability and Health-Children and Youth
IPP: Interprofessional practice
PACTR: Pan African Clinical Trial Registry
WHO: World Health Organisation
